# Using orthopaedic health care resources efficiently: A cost analysis of day surgery for unicompartmental knee replacement

**DOI:** 10.1016/j.knee.2024.06.006

**Published:** 2024-07-03

**Authors:** Takhona G. Hlatshwako, Cathy Jenkins, Sarah Wordsworth, David Murray, Karen Barker, Helen Dakin

**Affiliations:** University College, University of Oxford, High Street, Oxford, OX1 4BH, UK; Physiotherapy Research Unit, Nuffield Orthopaedic Centre, Windmill Road, Headington, Oxford, OX3 7HE, UK; Nuffield Department of Population Health, University of Oxford, Old Road Campus, Headington, Oxford, OX3 7LF, UK; Nuffield Department of Orthopaedics, Rheumatology and Musculoskeletal Sciences, Nuffield Orthopaedic Centre, Windmill Road, Headington, Oxford, OX3 7HE, UK; Physiotherapy Research Unit, Nuffield Orthopaedic Centre, Windmill Road, Headington, Oxford, OX3 7HE, UK; Nuffield Department of Population Health, University of Oxford, Old Road Campus, Headington, Oxford, OX3 7LF, UK; a Nuffield Department of Population Health, University of Oxford, UK; b Physiotherapy Research Unit, Nuffield Orthopaedic Centre, Oxford University Hospitals NHS, UK; c Oxford NIHR Biomedical Research Centre, UK; d Nuffield Department of Orthopaedics, Rheumatology, and Musculoskeletal Sciences, University of Oxford, UK

**Keywords:** Partial knee replacement, unicompartmental knee arthroplasty, day surgery, length of stay, cost analysis, enhanced recovery after surgery.

## Abstract

**Background:**

Day surgery for unicompartmental knee replacement (UKR) could potentially reduce hospital costs. We aimed to measure the impact of introducing a day surgery UKR pathway on mean length of stay (LOS) and costs for the UK NHS, compared to an accelerated inpatient pathway. Secondly, the study aimed to compare the magnitude of costs using three costing approaches: top-down costing; simple micro-costing; and real-world costing.

**Methods:**

We conducted an observational, before-and-after study of 2,111 UKR patients at one NHS hospital: 1,094 patients followed the day surgery pathway between September 2017 and February 2020; and 1,017 patients followed the accelerated inpatient pathway between September 2013 and February 2016. Top-down costs were estimated using Average NHS Costs. Simple micro-costing used the cost per bed-day. Real-world costs for this centre were estimated by costing actual changes in staffing levels.

**Results:**

532 (48.5%) patients in the day surgery pathway were discharged on the day of surgery compared with 36 (3.5%) patients in the accelerated inpatient pathway. The day surgery pathway reduced the mean LOS by 2.2 (95% CI: 1.81, 2.53) nights and was associated with an 18% decrease in Average NHS Costs (p<0.001). Mean savings were £1,429 per patient with the Average NHS Costs approach, £905 per patient with the micro-costing approach, and £577 per patient with the “real-world” costing approach. Overall, moving NHS UKR surgeries to a day surgery pathway could save the NHS £8,659,740 per year.

**Conclusion:**

Day surgery for UKR could produce substantial cost savings for hospitals and the NHS.

## Abbreviations

UKR: Unicompartmental knee replacement
NHS: National Health Service
(LOS): Length of stay
(NOC): Nuffield Orthopaedic Centre
(OUH): Oxford University Hospitals
(HRG): Healthcare Resource Group

## 1 Introduction

Hospital waiting lists have reached record highs in the United Kingdom (UK), with 1 in 8 people currently on a waiting list for the National Health Service (NHS) [1]. Orthopaedic treatments have the longest waiting lists, with about 800,000 patients awaiting care, half of whom have been waiting longer than the NHS target waiting time of 18 weeks [1].

Given increasing pressures on the NHS, there is an urgent need for clinical pathways that use healthcare resources efficiently. One potential solution is day surgery, which could free up hospital beds and staff, and potentially reduce waiting lists over time.

Unicompartmental knee replacement (UKR) may be particularly suitable for day surgery, as it is generally associated with a shorter hospital stay than total knee replacement, quicker recovery, and lower postoperative morbidity [3, 4]. In 2019, 6,060 UKR procedures were undertaken in NHS units in England, Wales and Northern Ireland, accounting for 9% of all knee replacements [5]. A growing body of evidence suggests that UKR can be safely offered on a same-day discharge basis [4, 6]. A meta-analysis showed that day surgery does not increase complication or readmission rates [4]. Moreover, in a patient satisfaction study, 90.5% of UKR day surgery patients expressed that they would choose a day surgery again over an inpatient procedure [7].

Although day surgery pathways are growing in popularity around the world [4], only 5.44% of UKR procedures in the UK NHS are done as day surgery [8]. Moreover, although previous studies have demonstrated the feasibility and safety of day surgery for UKR, there is currently no evidence on the cost impact of such pathways in the UK. A US-based study found that day surgery UKRs could save up to $20,500 per procedure compared to inpatient procedures [9]. Previous studies also focussed on selected cohorts for day surgery, rather than including all UKR patients.

This study aimed to estimate the impact of a policy change from an accelerated inpatient pathway to a day surgery pathway for UKR on costs and mean length of stay (LOS) in the UK NHS. We used real-world data from a Specialist Orthopaedic Unit within an NHS Foundation Trust hospital setting. Furthermore, we sought to compare the magnitude of cost savings associated with the policy change using three different costing methods relevant to different stakeholders in the healthcare system: a top-down costing approach estimating cost savings from the NHS perspective; a simple micro-costing approach estimating cost savings from the hospital perspective; and the ‘real-world’ cost savings actually realised by a single centre following the policy change.

## 2 Methods

### 2.1 Study design and population

We conducted a retrospective before-and-after study of a policy change at the Nuffield Orthopaedic Centre (NOC) within the Oxford University Hospitals (OUH) NHS Foundation Trust, Oxfordshire, UK. The medial or lateral Oxford® Partial Knee (Zimmer Biomet) was used for all surgery.

Prior to September 2016, patients followed the accelerated inpatient pathway of early commencement of knee flexion and mobilisation (described previously [10]). Patients would spend a night or more in hospital following surgery but were always discharged as soon as they were medically fit and mobile. The day surgery pathway (described previously [6] and in supplementary material) differed in that all patients listed for UKR surgery were treated with the intention of same-day discharge. They mobilised early but delayed any knee flexion until day 5 to 7 when those discharged on the day of surgery or day 1 returned to hospital for a change of dressing and commenced physiotherapy exercises. The pathway was designed to be non-selective and flexible (regardless of age and medical conditions). However, patients who could not be discharged on the day of surgery would stay overnight until they were medically fit and safely mobile. When the day surgery pathway was introduced, physiotherapy clinics and shift patterns were reorganised to facilitate same-day discharge [6].

**Figure 1:**
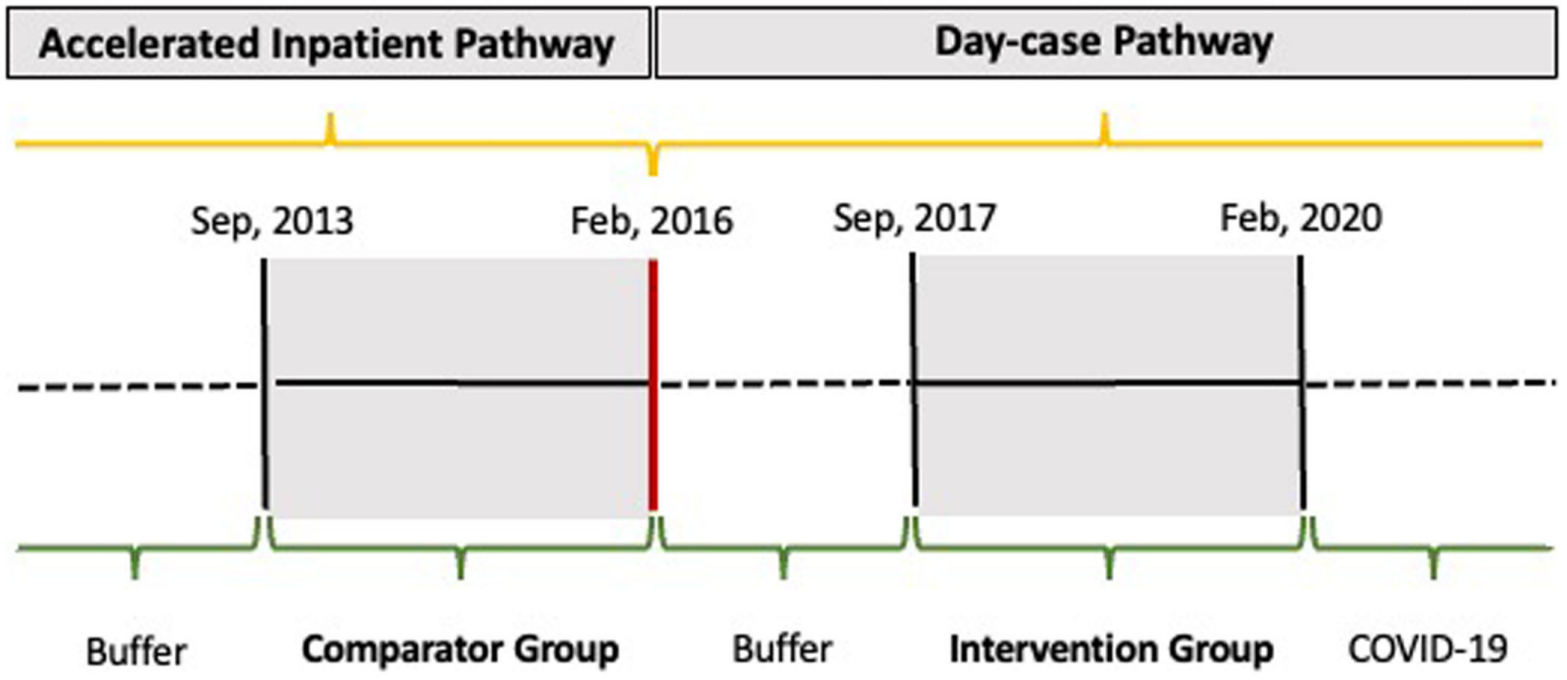
Timeline of Accelerated Inpatient Pathway and Day surgery Pathway at the Nuffield Orthopaedic Centre. Figure shows the timeline of the accelerated inpatient patient pathway and day surgery pathway and the dated selection of the comparator and intervention groups.

The comparator (accelerated inpatient pathway) group comprised all patients who underwent UKR at the NOC between September 1, 2013, and February 29, 2016, before the protocol change. The intervention (day surgery pathway) group comprised all patients undergoing UKR at NOC between September 1, 2017, and February 29, 2020, after the day surgery protocol was introduced. The first year of the day surgery protocol was excluded from our analysis to focus on the period when the pathway was fully adopted. Data after February 2020 were excluded (Figure 1) to remove the effects of the COVID-19 pandemic on case-mix. The study populations were unselected: i.e., all patients who received UKR during each study period went through the same protocol and all were included in the analyses. Our only inclusion criterion was undergoing UKR at NOC in one of the relevant date ranges. This reduced the risk of bias and ensured that the patients were representative of clinical practice.

### 2.2 Data sources

Oxford University Hospitals (OUH) NHS Foundation Trust provided patient-level data on the comparator and intervention groups that had been sent to Hospital Episode Statistics (HES), which collects details on admissions at NHS hospitals in England. Descriptive data on reasons for delayed discharge (≥1 night in hospital) were obtained by clinical audit of electronic patient records at the NOC after the policy change. Reasons for delayed discharge were only available for 463 people in the intervention group. Data on primary care consultations, medication, follow-up, rehabilitation and readmission were unavailable. However, the post-discharge rehabilitation and follow-up protocols were the same from day 5-7 onwards for both pathways and patients were discharged with the same medication package, so we did not expect these to differ between day surgery and inpatient pathways. Data on complications and comorbidities were available in the HES extract. However, due to the nature of coding, any complications that may have occurred during or after surgery were not distinguishable from existing comorbidities. As such, these data were not used in the primary analysis and reserved only for the sensitivity analysis. We were unable to obtain unbiased data on readmissions to hospital as data on readmissions to hospitals other than OUH were not available. Additionally, approximately 30% of patients undergoing joint replacement at the NOC live outside Oxfordshire. Moreover, a meta-analysis indicates that the rate of readmissions between the day surgery pathway and inpatient pathway is not significantly different [4]. We therefore used a literature review to estimate a readmission rate for the sensitivity analysis.

The study was approved on May 24, 2023, by the OUH NHS Foundation Trust Integrated Governance System as a clinical audit not requiring ethical approval (Ulysses Number 8278).

### 2.3 Costing methods

In order to present the cost savings relevant to the perspectives of different stakeholders, we compared three costing approaches: Top-down costs based on Average NHS Costs – previously known as NHS Reference Costs [11] – (NHS perspective); simple micro-costs based on bed-day costs (hospital perspective); and “real-world cost savings” realized by the centre (NOC perspective). The reference year was 2021-22 for all costs in both groups to ensure differences in costs were not affected by inflation. Discounting for time preference [12] was not applied as the time horizon for all analyses was one week or hospital discharge date. The Supplementary Material details the specific costs used and further methods.

The National Institute of Health and Care Excellence (NICE) recommends Average NHS Costs as a source of unit costs for economic evaluation [13]. Average NHS Costs [14] provide the mean cost of admissions for each Healthcare Resource Group (HRG): groups of comparable procedures that have similar cost from the NHS perspective. Patients who were discharged on the day of surgery were assigned the day-case HRG cost, and others were assigned the inpatient HRG cost. For patients discharged on the day of surgery or day 1 in the day surgery pathway group, we added on the cost of one hospital physiotherapy consultation, which is done 5-7 days post-operatively as part of this protocol. We also applied the cost of one hospital physiotherapy consultation to patients in the accelerated inpatient pathway group who were discharged on the day of surgery, reflecting clinical practice at that time.

For the bed-day cost approach, the incremental cost per night spent in hospital (excluding surgery) was based on the NHS excess bed-day Average NHS Cost for very major knee procedures. The bed-day cost represents the cost of a single night spent in hospital, therefore more closely reflects the actual costs incurred by a hospital at a micro-level, whereas the Average NHS Cost is an average of costs incurred by the NHS at a national level. The bed-day cost was applied for each subsequent night spent in hospital. For day surgery patients, only the day 5-7 physiotherapy cost was applied as these patients do not incur bed-day costs.

In practice, the cost savings that can be realised by an individual hospital do not necessarily equal the difference between HRGs or the mean cost of bed-days saved. Hospitals must generally employ a certain number of doctors and nurses and pay overheads and staff costs on an integer number of ward bays. We therefore compared the costs that would be estimated by economic evaluations (using the NHS or hospital perspective) with the “real-world cost savings” that the NOC was able to realise through the policy change. We included the savings from reduced nursing staff and the cost of additional physiotherapists required to implement the day surgery pathway. We included salaries and on-costs but excluded overheads (as these were not materially affected by the policy change) and staff qualifications costs (as these are not incurred by the hospital). Although there are likely to be modest savings for lighting, linen and meals, these were conservatively excluded due to data availability.

To estimate the financial impact at a national level of the policy change, we carried out a budget impact analysis by multiplying the mean savings from the day surgery pathway by the total number of NHS UKRs performed in England, Wales, and Northern Ireland in 2019 according to the National Joint Registry [5]. We used 2019 data as subsequent years were impacted by the COVID-19 pandemic.

### 2.4 Statistical analysis

Multivariable regression was conducted to control for age and sex, and to cluster by the consultant performing the procedure. We used two-part models to allow for the skewed distribution of LOS and patients who were discharged on the day of surgery. The first part of the model used logistic regression to model the probability of being discharged on the day of surgery, and the second part utilized a generalized linear model to estimate the number of nights spent in hospital among inpatients.

Sensitivity analyses tested the robustness of results. First, we added complication/comorbidity scores as a covariate. This variable was not in the base-case model as it includes any complications that may have occurred during or after surgery as well as pre-existing comorbidities and could therefore bias the results. A second sensitivity analysis assessed the impact that readmissions could have on costs by conservatively assuming that 2% more people would be readmitted for two nights under the day surgery pathway but not the inpatient pathway, which was based on the absolute readmission rates observed among day surgery patients in previous studies (0.88% to 3.72% [15, 16]). A meta-analysis showed that readmission rates for day surgery pathways tend to be similar to inpatient pathways [4], so this analysis should be considered conservative.

All analyses were two-tailed with a 0.05 significance level. All statistical analyses were conducted in STATA software (versions 17 and 18, Stata Corp, College Station, Texas).

## 3 Results

### 3.1 Patient characteristics

The study sample included 2,111 patients: 1,017 (48%) underwent UKR through the inpatient pathway from September 1, 2013 to February 29, 2016 (comparator group), and 1,094 (52%) underwent UKR through the day surgery pathway from September 1, 2017 to February 29, 2020 (intervention group). Eleven of the 16 consultants performing procedures operated in both time periods. Patients were aged between 23 and 97 years, with a mean age of 67 years and no significant difference in age or sex between groups (Table 1).

In both study groups, on average, patients had waited >100 days for their UKR procedure. The waiting time was higher in the later study period, reflecting increasing waiting time trends nationally [17]. Additionally, slightly more patients in the intervention group (47.2%, 516/1094) had >1 recorded comorbidity/complication than the comparator group (34.7%, 353/1017; Table 2). However, this difference is likely to be due to changes in the NHS guidelines for clinical coding of comorbidity/complication scores between the two study periods [18].

### 3.2 Length of stay

**Figure 2:**
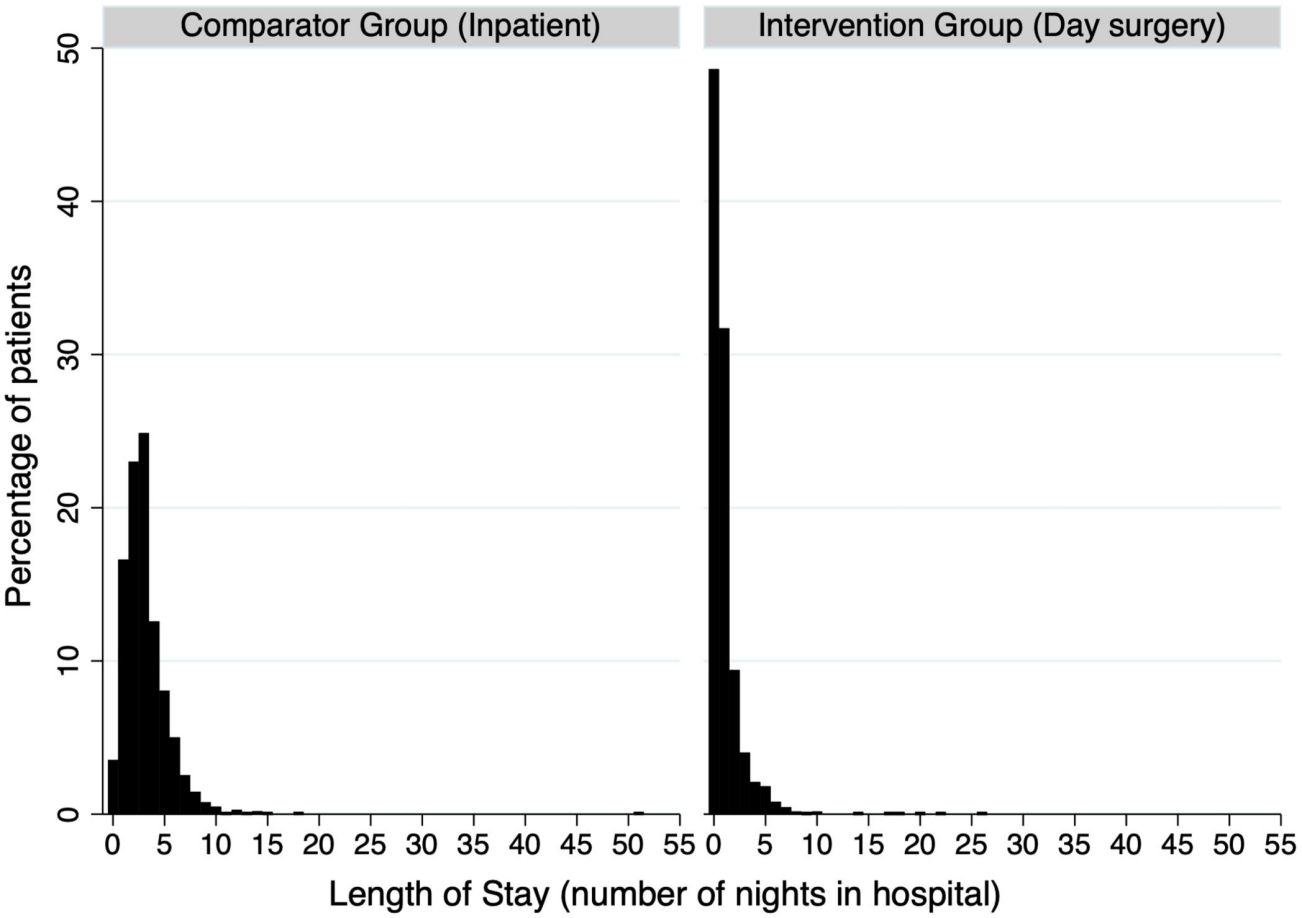
Histogram of length of stay by clinical pathway. (Left) Length of stay by number of nights spent in hospital in the comparator group. (Right) Length of stay by number of nights spent in hospital in the intervention group. * There was one patient in the comparator group with an LOS >50 days.

There were 36 patients (3.5%) in the comparator group who were discharged on the day of surgery compared to 532 patients (48.5%) in the intervention group (Table 3, Figure 2). The main reasons for delayed discharge included reduced sensation/muscle strength in leg, medical concerns, dizziness/nausea, pain, and social reasons (e.g. living alone, Table 4).

The odds of being discharged on the day of surgery, adjusting for age and sex, were 27.8 (95% confidence interval [CI]: 10.7, 72.3) times higher in the intervention group compared to the comparator group (p<0.001). Among patients who were not discharged on the same day, the mean LOS was 41% (95% CI: 32%, 49%) lower in the intervention group compared to the comparator group (p<0.001; Table 5). Combining both models, the adjusted mean LOS in the comparator group was 3.19 (95% CI 2.85, 3.53) nights, compared with 1.02 (95% CI: 0.90, 1.13) nights in the intervention group. Overall, shifting from the accelerated inpatient pathway to the day surgery pathway was associated with 2.17 (95% CI: 1.81, 2.53) fewer nights spent in hospital.

### 3.3 Costs and cost savings

**Figure 3:**
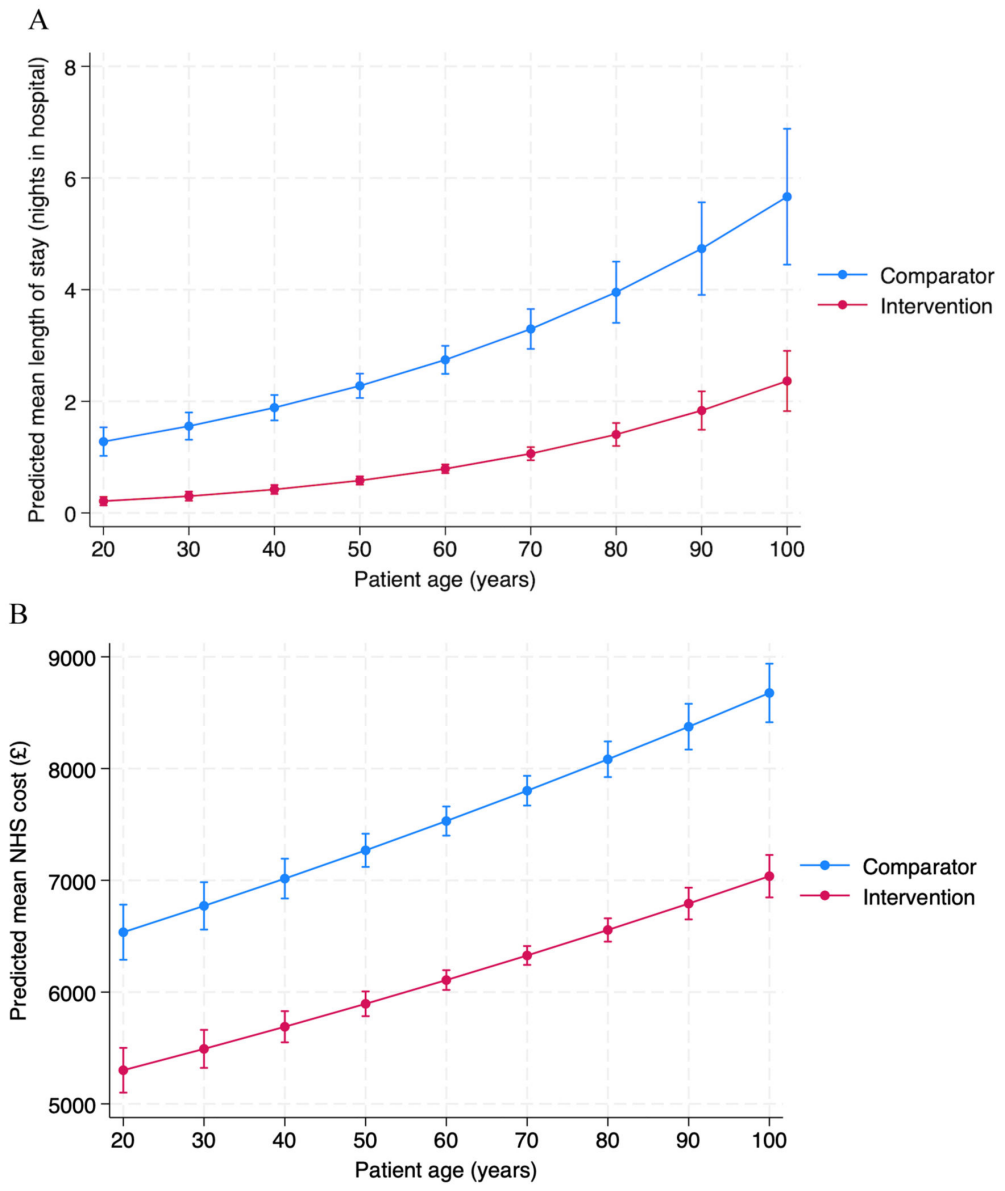
Mean length of stay and mean NHS cost by patients’ age. (A) Mean length of stay for patients of different ages in the comparator and intervention group. (B) Predicted mean NHS costs for patients of different ages in the comparator and intervention group. Values are adjusted for sex and consultant. Error bars show 95% confidence intervals around predicted values. Length of stay and costs increase with age in both groups, however, both length of stay and costs are lower in the intervention group (day surgery pathway) at all ages.

Under the Average NHS Costs approach (NHS perspective), the model estimated a 18% (95% CI: 16%, 20%) decrease in total costs per patient in the intervention group compared to the comparator group (p<0.001; Table 6). Under the bed-day costs approach (hospital perspective), the intervention group was associated with a 63% (95% CI: 58%, 68%) decrease in bed-day costs compared to the comparator group (p<0.001; Table 6). The absolute cost saving was £1,429 (95% CI: £1252, £1,607) per patient using the Average NHS Cost approach and £905 (95% CI: £733, £1,078) per patient using the bed-day costs approach. The day surgery pathway was associated with a lower mean LOS and costs for all ages compared to the inpatient pathway (Figure 3).

Shifting from the inpatient pathway to the day surgery pathway nationally could potentially save the NHS in England, Wales, and Northern Ireland about £8.7 million per year from the NHS perspective. Using the bed-day costs approach, the potential savings would be around £5.5 million per year (Table 7).

The introduction of the UKR day surgery pathway enabled the NOC to close one four-bed bay on the post-surgical ward. This required one less nurse and one less healthcare assistant, saving £6,898 per week (Table 8). The day surgery pathway required an additional physiotherapy shift in the early evening after surgery and an additional physiotherapy visit on day 5-7, costing £1,891 per week. The net staff savings to NOC were £250,370 per year, or £577 per patient.

### 3.4 Sensitivity analyses

Adding comorbidities/complications into the model estimating LOS slightly increased the odds ratio for same-day discharge from 27.8 (95% CI: 10.7-72.3) to 32.4 (95% CI: 13.2-79.9; Supplementary Material, Tables S4-S5). Adding comorbidities/complications into the model predicting costs (by either approach), had minimal impact.

When we conservatively assumed that readmissions were higher with the day surgery pathway, the cost savings using the bed-day costs approach decreased slightly, although costs remained £885 per patient lower with this pathway overall (p<0.001; Supplementary Material, Tables S6-S7).

## 4 Discussion

As the NHS and other healthcare systems grapple with increasing demand for care and longer waiting lists for orthopaedic treatments, day surgery pathways present a feasible option to improve access to joint replacement surgery and potentially reduce waiting lists over time. We found that when the day surgery pathway for UKR was introduced at an NHS Specialist Orthopaedic Unit, the mean LOS reduced by 2.2 nights. The day surgery pathway for UKR was associated with lower mean costs than the traditional inpatient pathway. Additionally, whilst 48.5% of patients were discharged on the day of surgery (a substantial improvement from 3.5% in the previous pathway), the mean LOS among inpatients was also reduced by 41% compared to the previous pathway. Reduced LOS and costs were seen for patients of all ages. The main reasons why patients were not discharged on the day of surgery included reduced sensation/muscle weakness, medical concerns (such as blood pressure issues or a bleeding wound) and social reasons (such as living alone). In addition to reducing staffing costs, the introduction of UKR day surgery provided additional capacity to do more orthopaedic operations.

The day surgery pathway was also associated with potential cost savings. From an NHS perspective, the day surgery pathway could save up to £8.7 million per year nationally. At the centre in this study, this pathway produced cost savings of about £392,770 per year (hospital perspective) in bed-day costs, of which £250,370 per year could be realised in practice (real-world cost savings). Average NHS Costs for inpatient knee procedures may overestimate the cost of UKR with an accelerated inpatient pathway due to variations in hospital protocols and the mix of procedures included in the same HRG code. Bed-day costs may more closely reflect the actual costs that the average hospital incurs in caring for patients. As such, the estimated cost-savings based on the bed-day cost approach may be regarded as more realisable cost savings for the average hospital from this policy change. Cost savings for other hospitals may be larger or smaller depending on patient numbers, ward set-up, fixed costs and staffing levels. For instance, after the NOC introduced the day surgery pathway, they were able to realise savings of £250,370 per year (or £577 per patient) by reducing the number of nursing posts. This demonstrates a real-world example of healthcare resources being optimised and redeployed for other hospital operations as needed.

The 55% difference in cost savings between costing approaches also has implications for the generalisability of the results to other settings and how the cost information could be used to help inform other health economic studies. The National Tariff [19], which reflects the amount hospitals are paid for each procedure or admission, is sometimes used for costing procedures in economic evaluation. However, this applies the same cost to day surgery and inpatient surgery, so using it in our costing analysis would have suggested no difference in costs between the two pathways. Although Average NHS Costs are widely recommended for costing hospital admissions [13], care should be taken to review the mix of indications included in HRGs and to consider micro-costing key cost drivers, particularly when the intervention changes costs within a hospital admission.

This study had several strengths. Firstly, both comparison groups included >1,000 unselected patients in each group. Other similar studies had smaller cohorts: for example, in one UK-based study only 72 patients met the inclusion criteria for day surgery [20]. Patients in our study were not pre-selected for either pathway, making the findings less biased, more time efficient to implement and more generalizable to other settings and patient populations. We are not aware of any previous studies with no pre-selection of patients or any that evaluate a fully-inclusive day surgery UKR pathway. Our study reduced the risk of bias by comparing all patients undergoing UKR in 2013-16 with all those undergoing UKR in 2017-2020. Clinical protocols used in some previous studies pre-selected day surgery patients based on preoperative fitness, or planned for some patients with comorbidities to stay overnight [20, 21]. On the contrary, the NOC protocol evaluated in our study used the same pathway for all UKR patients, which is more equitable and gives all patients the same opportunity for early discharge. Another strength of our study was the use of appropriate regression models to estimate LOS reduction and cost-savings.

The main limitation of this study was the lack of randomisation. Although we adjusted for age and sex and evaluated the impact of adjusting for comorbidities/complications, there may have been other differences between the two pathways that were not measured or adjusted for. Another limitation was the lack of data on readmissions. However, a meta-analysis of day surgery pathways versus inpatient pathways found that there was no statistically significant difference in readmission rates [4]. Moreover, our sensitivity analysis found that the day surgery pathway would still have significantly lower costs than the inpatient pathway even if 2% more day surgery patients were readmitted.

This study demonstrates that there are likely to be substantial cost savings if a day surgery pathway for UKR is introduced nationally into clinical care. This pathway may also improve access to knee replacement and other orthopaedic procedures by freeing up healthcare resources, reducing waiting lists, and producing cost savings that may be redirected towards other healthcare priorities. Although unit costs vary between countries and settings, the general finding that UKR day surgery saves money is likely to have general relevance for other countries.

## Supplementary Material

Supplementary material

## Figures and Tables

**Table 1:** Patient characteristics by clinical pathway.

Characteristic	Intervention group (n = 1094)	Comparator group (n = 1017)	*P*-value*
**Age** (years),			
Mean (SD)	67.6 (10.6)	66.9 (10.3)	0.133
Range (IQR)	23 - 95 (15)	32 – 97 (14)	
**Sex**, n (%)			0.652
Male	511 (46.7)	485 (47.7)	
Female	583 (53.3)	532 (52.3)	
**Time on waiting list** (days),			
Mean (SD)	127.3 (62.5)	100.9 (43.0)	<0.001

* Based on two-sample Student’s *T* test for continuous variables and Chi-square test for categorical variables.

**Table 2:** Comorbidity and complication scores of study participants.

Characteristic	Intervention group (n = 1094)	Comparator group (n = 1017)	*P*-value
**Comorbidity/complication (CC) score** (Healthcare Resource Group)*, n (%)			0.001**
CC score 0-1	578 (52.8)	664 (65.3)	
CC score 2-3	355 (32.4)	270 (26.5)	
CC score 4-5	111 (10.2)	61 (6.0)	
CC score 6-7	39 (3.6)	16 (1.6)	
CC score 8+	11 (1.0)	6 (0.6)	

* Based on Healthcare Resource Group (HRG4+) classification of Hospital Episode Statistics.

** Based on Chi-square Test of association.

**Table 3:** Type of admission and unadjusted mean length of stay.

	Intervention Group (n=1017)	Comparator Group (n=1094)	*P-value*
Type of admission*, n (%)			<0.001*
Same-day discharge	532 (48.5)	36 (3.5)	
Inpatient	564 (51.5)	981 (96.5)	
**Unadjusted length of stay (days)**, mean (Standard Deviation)	1.03 (1.96)	3.16 (2.58)	<0.001**

* Based on Chi-square test of association.

** Based on two-sample Student’s T test.

**Table 4:** Reasons for delayed discharge among patients who underwent UKR after the introduction of the day surgery pathway who were not discharged on the day of surgery.

Reason for delayed discharge	N (%)
Reduced sensation/muscle strength	90 (19.4)
Medical concerns*	90 (19.4)
Major medical concerns	1 (0.2)
Nausea & vomiting/dizzy	61 (13.2)
Pain	43 (9.3)
Slow mobilisation	38 (8.2)
Lives alone/social reasons	35 (7.6)
Not seen by physiotherapist on the day of surgery	23 (5.0)
Planned overnight stay	18 (3.9)
Operation note instructions	14 (3.0)
“Other” reasons**	50 (10.8)
Total number of delayed discharges	463

* Examples of medical concerns: required sedation in recovery; anxiety; monitored in recovery overnight; comorbidities; bleeding/oozy wound; raised temperature; oxygen desaturation on mobilisation; dementia; blood pressure or cardiac issues; migraine.

** Examples of other reasons: late back from theatre; patient unwilling to mobilise.

**Table 5:** Two-part model results for length of stay.

Variable	Part I*: Odds Ratio for same day discharge (95% CI)	P-value	Part II**: LOS Ratio*** (95% CI)	P-value
**Clinical pathway**				
Comparator group	Reference		Reference	
Intervention Group	27.8 (10.7-72.3)	<0.001	0.59 (0.51-0.68)	<0.001
**Sex**				
Female	Reference		Reference	
Male	0.14 (0.05-0.43)	<0.001	0.86 (0.79-0.94)	<0.001
**Age** (years)	0.98 (0.97-0.98)	<0.001	1.01 (1.01-1.02)	<0.001

* Part I of the model was logistic regression, adjusted for age and sex and clustered by consultant.

** Part II of the model was a generalised linear model with Poisson distribution and log link, adjusted for age and sex and clustered by consultant.

*** Ratios are obtained by taking the exponent of the coefficient output of the regression model.

**Table 6:** Generalized linear model results for costs.

Variable	Cost Ratio* by Average NHS Costs approach (95% CI)	P-value	Cost ratio* by bed-day costs approach (95% CI)	P-value
**Clinical pathway**				
Comparator group	Reference		Reference	
Intervention Group	0.82 (0.80-0.84)	<0.001	0.37 (0.32-0.42)	<0.001
**Sex**				
Female	Reference		Reference	
Male	0.97 (0.95-0.99)	0.005	0.79 (0.70-0.88)	<0.001
**Age** (years)	1.00 (1.00-1.01)	<0.001	1.02 (1.01-1.02)	<0.001

* Ratios are obtained by taking the exponent of the coefficient outputs of the regression model. A cost ratio of 0.82 equals a 18% reduction in costs.

**Table 7:** Estimated cost savings per year using each costing method.

	Inpatient pathway	Day surgery pathway	Difference: net savings (95% CI)
**Average NHS Costs approach:**			
Mean total cost per patient*	£7,732	£6,303	£1,429 (£1252, £1,607)
No. of UKRs performed nationally per year**	6,060	6,060	-
**Total national cost**	**£46,855,920**	**£38,196,180**	**£8,659,740** **(£7,587,120, £9,738,420)**
No. of UKRs performed at NOC per year**	434	434	-
**Total NOC cost**	**£3,355,688**	**£2,735,502**	**£620,186 (£543,368, £697,438)**
**Bed-day costs approach:**			
Mean bed-day cost per patient*	£1,432	£527	£905 (£733, £1,078)
No. of UKRs performed nationally per year**	6,060	6,060	-
**Total national cost**	**£8,677,920**	**£3,193,620**	**£5,484,300** **(£4,441,980, £6,653,680)**
No. of UKRs performed at NOC per year***	434	434	-
**Total NOC cost**	**£621,488**	**£228,718**	**£392,770** **(£318,122, £467,852)**

* Estimated mean predicted by GLM model, controlling for age, gender and clustered by consultant.

** Number of UKRs performed in the NHS in England, Wales, Northern Ireland in 2019 from the National Joint Registry [5].

*** Actual number of UKRs performed in 2019 at the Nuffield Orthopaedic Centre based on our audit data.

**Table 8:** Estimated real-world cost savings per year (2021-2 UK pounds).

Staff role	Annual salary [22, 23]	Salary on costs [22]	Hourly cost*	Hours/day role is required	Days/week that role is required	Incremental cost/week from day case UKR
**Savings on nursing costs**						
Band 6 nurse	£36,415	£11,017	£30.54	24	6	-£4,398.07
Band 3 health-care assistant	£21,053.50	£5,909.74†	£17.36	24	6	-£2,500.13
**Additional physiotherapy costs**						
Band 6 physiotherapist providing pre-discharge care	£36,051	£10,894	£29.10	7.5	5	£1,091.40
Band 7 physiotherapist for early outpatient review	£43,793	£13,515	£35.53	7.5	3	£799.40
**TOTAL savings for Nuffield Orthopaedic Centre per week**						**-£5,007.40**
**TOTAL savings for Nuffield Orthopaedic Centre per year**						**£250,370.09**
**Total savings per patient treated (assuming 434 patients treated during 50 weeks of operations)**						**£576.89**

* Hourly costs were estimated by summing the annual salary and salary on costs and dividing by the number of working hours per year. We used published estimates of 1533 working hours per year for nurses and healthcare assistants and 1633 working hours per year for physiotherapists [22].

† Annual salary obtained from NHS pay scales [23]. The ratio of on costs to salary was assumed to be the same as for band 4 nurses.

## Data Availability

The costing models used in this paper are available from the corresponding author on request. Hospital Episode Statistics data can be obtained from NHS England at https://digital.nhs.uk/data-and-information/data-tools-and-services/data-services/hospital-episode-statistics.
