# Supplementary material for: Using orthopaedic health care resources efficiently: A cost analysis of day surgery for unicompartmental knee replacement
Source: Knee. Author manuscript; Available in PMC 2024 Jul 17. (PMC7616244; doi:10.1016/j.knee.2024.06.006)
Supplement: Supplementary material [file EMS197332-supplement-Supplementary_material.docx]

**Using orthopaedic health care resources efficiently: A cost analysis of day surgery for unicompartmental knee replacement.**

**Supplementary methods**

**Day surgery protocol**

The day surgery pathway is described in detail in Jenkins et al. [[1](#_ENREF_1)] and summarised briefly in this section. Upon admission, unicompartmental knee replacement (UKR) patients underwent assessments by a nurse, surgeon, and anaesthetist. Most cases were medial, with cementless fixation and mobile bearings. On the lateral side cemented components were used with both fixed and mobile bearings. A standard minimally invasive surgical technique was used for all patients, typically under general or spinal anaesthesia with peri-articular local infiltration, discouraging the use of femoral and sciatic nerve blocks. Wound drains were never used. Post-surgery, patients were encouraged to promptly resume eating and drinking and were seen by a physiotherapist for sensation and motor power assessment. Patients were instructed to keep their knee straight and mobilised weight bearing as tolerated with crutches. Instruction was given to keep their compression bandage intact and maintain knee extension until they returned to a designated UKR clinic 5 to 7 days later.

Discharge criteria included satisfactory X-ray and surgical team assessment, along with guidance on post-discharge care, pain management, and emergency contact information. Patients unable to return home on the day of surgery were discharged the following day if medically feasible, with similar instructions.

Those discharged on days 0 or 1 returned to the UKR clinic around the fifth post-operative day. Outpatient physiotherapy was arranged as clinically indicated for range of motion improvement but not routinely provided. Further follow-up at six weeks included surgical team or specialist physiotherapist evaluation and completion of satisfaction surveys, which was common to both the day case and inpatient pathways.

The accelerated pathway used prior to 2016 is described by Jenkins et al. 2006 [[2](#_ENREF_2)].

**Costing methodology**

*Average NHS costs approach (NHS perspective)*

Average NHS Costs (also known as NHS Reference Costs) were used to quantify the total costs associated with each UKR pathway. This follows a top-down approach, where a total budget is assigned to particular healthcare services using routinely available data to give average costs. Average NHS Costs are schedules of the average unit cost of healthcare services published by the NHS [[3](#_ENREF_3)]. Average NHS Costs give the average cost from the perspective of the NHS, as this method estimates the average cost of healthcare services across all NHS providers.

When a clinical episode requiring a healthcare service is initiated, a patient’s clinical information is “coded” using ICD-10 codes (International Classification of Diseases) and OPCS Codes (Classification of Interventions and Procedures). ICD-10 codes are used to describe the diagnoses of the patient, and OPCS codes are used to describe the treatment/procedure rendered to them. These codes are recorded in Hospital Episode Statistics (HES) and assigned a Healthcare Resource Group (HRG).

HRG codes represents a group of similar treatments that utilize a similar amount of healthcare resources. An HRG Code is made of five characters. The first four characters are known as the root of the HRG code, representing the class of diseases and/or intervention received by the patient. For instance, for the HRG Code “HN22E,” the first two characters “HN” represent “Musculoskeletal Conditions.” The digits “22” represent “Very Major Knee Procedure for Non-trauma.” The fifth letter of an HRG Code represents the complications or comorbidities’ score (CC score) of the patient, which are determined by ICD-10 codes and labelled “A to E” by decreasing severity. For instance, for the HRG Code “HN22E” the letter “E” indicates that the patient has 0-1 complications or comorbidities. There are separate reference costs for each letter of the CC score.

The two study periods being analysed were governed by different HRG coding guidelines: HRG4 in the comparator group, and HRG4+ in the intervention group. We therefore used the HRG4+ 2021/22 National Costs Grouper Software to produce the most current codes for the comparator group [[4](#_ENREF_4)]. The Grouper software utilized ICD-10 codes and OPCS procedure codes of each patient to determine how they would have been categorized in HRG4+, therefore allowing the two study periods to be comparable.

Average NHS Costs present the mean NHS cost for each HRG. Hospitals are reimbursed for each admission or procedure by the patient’s Integrated Care Service based on the “payments by results” tariff for each HRG [[5](#_ENREF_5)]. The “payment by results” tariff is based on previous years’ average costs, although it incorporates incentives for efficiency. The Payment by Results tariff does not differentiate between day-case and inpatient procedures, and reflects the cost to Integrated Care Services rather than to the NHS as a whole, so was not used in this analysis.

In the dataset used for this analysis, each patient observation had an HRG code attached to it that described the procedure the patient received during their clinical episode. A “total average cost” variable was created by inputting the cost associated with a patient’s HRG code (sourced from the schedule of Average NHS Costs), and the assigned cost was conditional on whether the patient was treated as a day-case or an inpatient (Table S1). The HRGs that cover UKR (HN22A-E) also encompass other types of surgery, including TKR and certain types of revision surgery. Average NHS Costs also reflect the national average cost of admissions, including centres that may have longer LOS than the NOC. The Average NHS Costs for UKR include the knee implant, surgeon time, ward time, overheads and physiotherapy.

In the day surgery pathway group, patients discharged on the day of surgery or day 1 were additionally assigned a physiotherapy cost as this cost is not included in the day-case HRG and is needed in this clinical protocol (see above). In the inpatient pathway group, we assumed that any patients who were discharged on the day of surgery would require one hospital physiotherapy visit for follow-up, reflecting standard practice at that time. We assumed that no patients in either group required time in high dependency unit or intensive care.

The “total average cost” variable was then used as the dependent variable in the GLM model, and the independent variable was the clinical pathway undergone by each patient i.e., day surgery pathway or accelerated inpatient pathway. This approach was taken to provide an estimate of cost savings particularly relevant for national policymakers.

| Table S1: Unit costs used in the analysis (2021-2 UK pounds) | | | |
| --- | --- | --- | --- |
| Resource | **Data source** | **Unit cost** | **Reference** |
| Day-case | | | |
| Day-case – Very Major Knee Procedures for Non-Trauma, (Complication score 8+) | NHS Schedule of Costs, 2020-21; HRG Code: HN22A | £4,774.85 | [[3](#_ENREF_3)] |
| Day-case – Very Major Knee Procedures for Non-Trauma, (Complication score 6-7) | NHS Schedule of Costs, 2021-22; HRG Code: HN22B | £4,182.21 | [[3](#_ENREF_3)] |
| Day-case – Very Major Knee Procedures for Non-Trauma, (Complication score 4-5) | NHS Schedule of Costs, 2021-22; HRG Code: HN22C | £4,212.82 | [[3](#_ENREF_3)] |
| Day-case – Very Major Knee Procedures for Non-Trauma, (Complication score 2-3) | NHS Schedule of Costs, 2021-22; HRG Code: HN22D | £4,007.08 | [[3](#_ENREF_3)] |
| Day-case – Very Major Knee Procedures for Non-Trauma, (Complication score 0-1) | NHS Schedule of Costs, 2021-22; HRG Code: HN22E | £4,427.97 | [[3](#_ENREF_3)] |
| Elective Inpatient | | | |
| Elective Inpatient – Very Major Knee Procedures for Non-Trauma, (Complication score 8+) | NHS Schedule of Costs, 2021-22; HRG Code: HN22A | £11,546.14 | [[3](#_ENREF_3)] |
| Elective Inpatient – Very Major Knee Procedures for Non-Trauma, (Complication score 6-7) | NHS Schedule of Costs, 2021-22; HRG Code: HN22B | £9,792.98 | [[3](#_ENREF_3)] |
| Elective Inpatient – Very Major Knee Procedures for Non-Trauma, (Complication score 4-5) | NHS Schedule of Costs, 2021-22; HRG Code: HN22C | £8,718.87 | [[3](#_ENREF_3)] |
| Elective Inpatient – Very Major Knee Procedures for Non-Trauma, (Complication score 2-3) | NHS Schedule of Costs, 2021-22; HRG Code: HN22D | £8,106.70 | [[3](#_ENREF_3)] |
| Elective Inpatient – Very Major Knee Procedures for Non-Trauma, (Complication score 0-1) | NHS Schedule of Costs, 2021-22; HRG Code: HN22E | £7,542.27 | [[3](#_ENREF_3)] |
| Other | | | |
| Physiotherapy session for 5-7 days after surgery | NHS Schedule of Costs, 2021-22 | £100 | [[3](#_ENREF_3)] |
| Bed-day cost | Estimated using 2017-2018 NHS Schedule of Costs: weighted average cost across HRGs HN22A-E, inflated to 2021-2 values. | £445 | [[6](#_ENREF_6), [7](#_ENREF_7)] |

*Bed-day costs approach (hospital perspective)*

A simple ‘bottom-up’ micro-costing method using bed-day costs was used. This approach used an estimate of the incremental cost per additional night spent in hospital that includes ward costs, meals and overheads but excludes theatre work up, anaesthetics, theatre time, components and immediate post-operative care. Up until 2017-2018, excess bed-day costs were presented in Average NHS Costs; these represented the additional cost of each 24-hour period spent in hospital beyond a trim point and are therefore a good proxy for the incremental cost of an additional night in hospital. This approach has been used to value bed-days in previous economic evaluations for knee replacement (e.g. Murray et al. [[8](#_ENREF_8)]) The 2017-2018 NHS cost schedule was used as this was the last available schedule that had data on excess bed-days as the NHS stopped reporting these data after 2018.

The cost/bed day used for the bed-day cost approach was therefore based on the weighted average cost per bed-day across HRGs HN22A-E in the year 2017-2018, [[6](#_ENREF_6)] weighted by number of excess bed days. The weighted average (Table S1) was adjusted for inflation from 2017/18 values to 2021/2 values using the NHS Cost Inflation Index (NHSCII) [[7](#_ENREF_7)].

The total bed-day cost equalled the LOS multiplied by the crude bed-day cost. Patients discharged on the day of surgery (i.e. those with zero LOS) — were assigned the physiotherapy cost (Table S1) instead of a bed-day cost (as they do not spend a night in hospital but return to the centre on day 5-7 after surgery for their physiotherapy appointment). Patients in the day surgery pathway who were discharged on day 1 were also assigned the physiotherapy cost as well as the cost of one night in hospital. The total bed-day cost was then used as the dependent variable in the GLM model, and the independent variable was the clinical pathway undergone by each patient. This approach was taken to provide an estimate of cost-savings particularly relevant for hospital administrators and local policymakers considering the day surgery pathway.

*Real-world costing (NOC perspective)*

We calculated the real-world cost savings realized by NOC through the reduction of nursing costs. The transition to the day surgery pathway meant that NOC required one less Band 6 nurse and one less Band 3 healthcare assistant. The savings on annual salaries were subtracted from the additional physiotherapy costs that NOC incurred during the policy change. These included salaries for a Band 6 physiotherapist treating patients before discharge and a Band 7 physiotherapist doing outpatient reviews at day 5-7. The net differences in salaries paid per year were interpreted as real-world cost savings. This approach was taken to provide an estimate of cost-savings particularly relevant for hospital administrators and clinicians at other centres considering the day surgery pathway.

**Statistical methodology**

As the LOS variable in this study was very right skewed and included many zeros (patients spending 0 days in hospital), a two-part model was chosen as the statistical method of analysis. In the first part of the model, logistic regression was used to determine the probability of same-day discharge i.e., the probability that a patient would be a day-case (LOS=0) in either pathway. In the second part of the model, a generalized linear model (GLM) was used to examine the magnitude of LOS among those who were not discharged on the same day of surgery (i.e., inpatients). The two-part model (twopm) command in STATA was used to estimate two-part models, which produced a combined estimate of effect based on both parts of the model. As LOS consisted of counts, the Poisson distribution with log link was used for this model. The choice of distribution family was tested using the Modified Parks Test.

GLM was chosen for the analysis of costs in this study as it does not impose normal distribution assumptions on the model variables. Particularly, exploratory data analysis showed that the cost variables in this study were non-normal and multimodal (Supplementary Figure S1, S2 and S3), thus GLM was deemed most appropriate. Moreover, a GLM model with the “log link” function eliminates the need for retransformation as inferences can be made on the original scale of the variable, thus this specification was chosen for the cost data in this analysis [[10](#_ENREF_10)].

Ordinary least squares (OLS) was not used in the base-case for either costs or LOS. GLM was chosen over OLS regression because these variables did not fit the assumption of normality or the assumption of constant variance. Moreover, as LOS included a substantial proportion of zero-values, fitting an OLS model on observations with non-zero values only could bias the results. Alternatively, OLS with log transformation could have potentially introduced retransformation bias.

In terms of interpretation, the coefficient output estimated by each model indicated the measure of the marginal effect of a one-unit change in the explanatory variable (i.e., changing from the inpatient pathway to the day surgery pathway) on the outcome variables (costs and LOS). The coefficients were interpreted as percentage changes by taking the exponent, subtracting one and multiplying by 100.

In the multivariable analysis, we adjusted for age and sex, and clustered by consultant to allow for correlations between groups of patients. The margins post-estimation command was then used to estimate between-group differences in length of stay and costs and confidence intervals around these differences.

**Qualitative analysis**

Additionally, a qualitative analysis of the “reasons for delayed discharge or return/readmission” was conducted for patients who were not discharged on the same day of surgery and/or returned after surgery in the intervention group. The reasons for delayed discharge or return/readmission were examined via a thematic analysis, which is a qualitative analytic method that categorizes major themes in non-quantitative data. Major themes were defined as reasons that came up most frequently in the data, and these reasons were identified through repeated reading of the data.

**Supplementary results**

**Exploratory data analysis**

Exploratory data analysis was conducted to determine the best fitting model for the variables in the dataset. The distributions of the main outcome variables, length of stay (LOS) and costs were explored using histograms. LOS was right-skewed, positive, and consisted of many zero values (Refer to figure in text).

Total Average NHS Costs were non-normal, multimodal, and positive (Figure S1). Bed-day costs were very right-skewed and positive (Figure S2).

*Figure S1. Histogram of total reference cost by clinical pathway.*


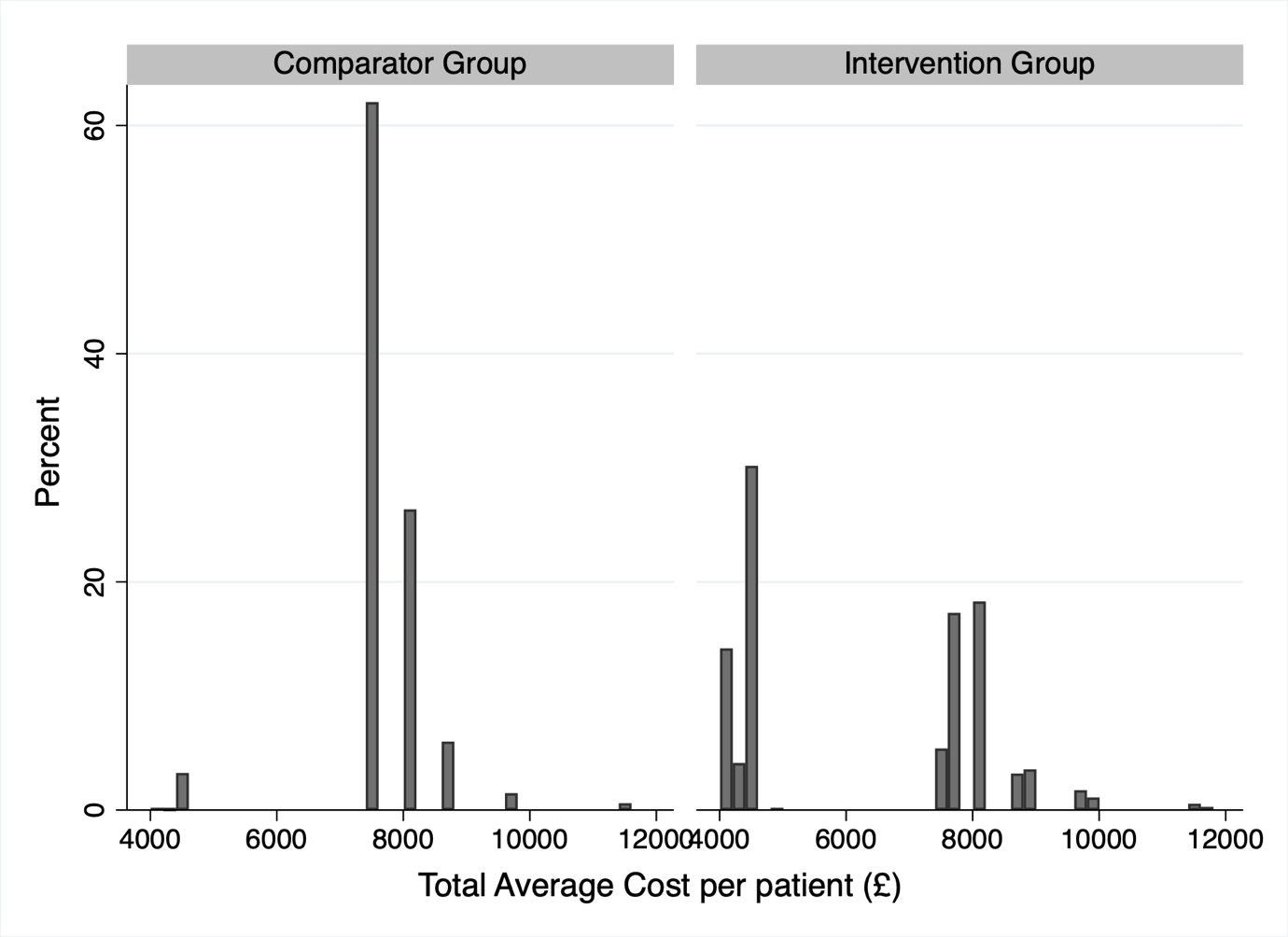


The non-normal distributions of the outcome variables suggested that ordinary least squares (OLS) regression would not be appropriate for the data as OLS assumes normal distribution. Furthermore, the length of stay variable included many zeroes, which further suggested that OLS with log transformation would also not be appropriate as the log of 0 is undefined. Therefore, generalized linear models were used in place of OLS.

*Figure S2. Histogram of bed-day cost by clinical pathway.*


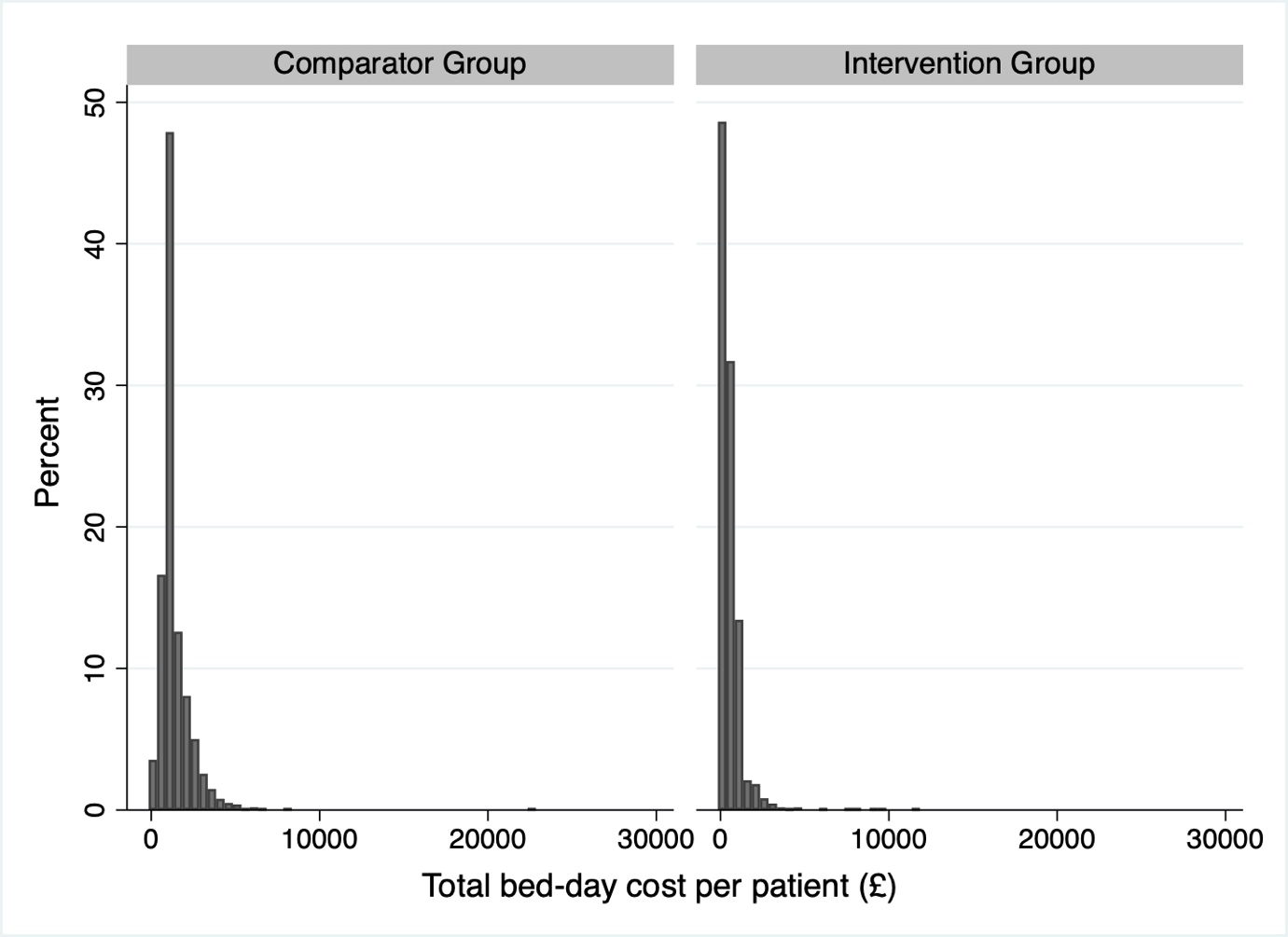


**Sensitivity analyses**

To test the robustness of the findings, sensitivity analyses were conducted with complications/comorbidities added as a covariate in both regression models on length of stay and costs. The effect of the added covariate on length of stay was notable in that the day-case odds ratio increased from 27.8 (95% CI: 10.7-72.3) to 32.4 (95% CI: 13.2-79.9; Table S4). This suggests that complications/ comorbidities have an effect on length of stay.

| Table S4: Length of stay regression results including comorbidities. | | | | |
| --- | --- | --- | --- | --- |
| Variable | **Part I:**  **Odds Ratio***  **(95% CI)** | **P-value** | **Part II:**  **LOS Ratio***  **(95% CI)** | **P-value** |
| Clinical pathway |  |  |  |  |
| Comparator group | Reference |  | Reference |  |
| Intervention Group | 32.4 (13.2-79.9) | <0.001 | 0.54 (0.47-0.62) | <0.001 |
|  |  |  |  |  |
| Age | 0.99 (0.98-1.00) | <0.013 | 1.01 (1.01-1.02) | <0.001 |
|  |  |  |  |  |
| Sex |  |  |  |  |
| Female | Reference |  | Reference |  |
| Male | 1.81 (1.56-2.11) | <0.001 | 0.81 (0.74-0.89) | <0.001 |
|  |  |  |  |  |
| Comorbidities/ Complications score |  |  |  |  |
| CC Score 0-1 | Reference |  | Reference |  |
| CC Score 2-3 | 0.56 (0.45-0.78) | <0.001 | 1.27 (1.18-1.37) | <0.001 |
| CC Score 4-5 | 0.35 (0.24-0.52) | <0.001 | 1.34 (1.17-1.55) | <0.001 |
| CC Score 6-7 | 0.21 (0.11-0.36) | <0.001 | 1.93 (1.61-2.31) | <0.001 |
| CC Score 8+ | 0.14 (0.04-0.29) | <0.002 | 3.82 (1.97-7.40) | <0.001 |
|  |  |  |  |  |
| *Ratios are obtained by taking the exponent of the coefficient output of the regression model. | | | | |

When comorbidities/complications were added to the generalized linear model on costs, the cost ratios did not change substantially for either the Average NHS costs approach or bed-day costs approach (Table S5). This suggests that both costing approaches were not too sensitive to the addition of comorbidities/complications. However, this finding may be biased by the manner in the which complications/comorbidities are coded in HES data; any complications that may have occurred during or after surgery are not distinguished from existing comorbidities, thus it is difficult to control for this variable adequately.

| Table S5: Generalized linear model results for costs including comorbidities. | | | | |
| --- | --- | --- | --- | --- |
| Variable | **Cost Ratio* by Average NHS costs approach (95% CI)** | **P-value** | **Cost ratio** by bed-day costs approach (95% CI)** | **P-value** |
| Clinical pathway |  |  |  |  |
| Comparator group | Reference |  | Reference |  |
| Intervention Group | 0.79 (0.78-0.81) | <0.001 | 0.34 (0.30-0.38) | <0.001 |
|  |  |  |  |  |
| Age | 1.00 (1.00-1.01) | <0.037 | 1.01 (1.00-1.02) | 0.001 |
|  |  |  |  |  |
| Sex |  |  |  |  |
| Female | Reference |  | Reference |  |
| Male | 0.95 (0.94-0.97) | <0.001 | 0.76 (0.68-0.85) | <0.001 |
|  |  |  |  |  |
| Comorbidities/ Complications Score |  |  |  |  |
| CC Score 0-1 | Reference |  | Reference |  |
| CC Score 2-3 | 1.09 (1.07-1.11) | <0.001 | 1.43 (1.27-1.61) | <0.001 |
| CC Score 4-5 | 1.22 (1.17-1.28) | <0.001 | 1.64 (1.33-2.01) | <0.001 |
| CC Score 6-7 | 1.44 (1.37-1.51) | <0.001 | 2.67 (2.16-3.32) | <0.001 |
| CC Score 8+ | 1.72 (1.54-1.91) | <0.001 | 4.05 (2.67-6.17) | <0.001 |
|  |  |  |  |  |
| *Ratios are obtained by taking the exponent of the coefficient outputs of the regression model. **Represents of the cost of staying in hospital. | | | | |

A second sensitivity analysis was conducted that conservatively assumed that readmission rates were 2% higher with the day surgery pathway than the inpatient pathway. Data on readmissions was not captured by the HES extract, therefore the readmission rate was assumed based on other studies found in a literature review. This 2% figure reflects the midpoint of the range of readmission rates reported with day case pathways in previous studies, which was from 0.88% to 3.72% [[11-13](#_ENREF_11)]. For the sensitivity analysis, it was assumed that every readmitted patient would spend two nights in hospital. The cost of readmission was calculated as double the bed-day cost (for the two-night stay). The randomselect command in Stata was used to randomly identify 22 patients in the day surgery pathway who were assumed to have been readmitted and accrue the cost of readmission. No patients in the comparator group were assumed to be readmitted since the aim of this analysis was to test cost differences if there were (hypothetically) a difference in readmission rates between pathways. The coefficients are shown with and without the effect of adding readmissions (Table S6). With readmissions, the day case pathway was associated with 62% lower costs than the inpatient pathway (using the bed-day approach), compared with 63% lower in the main analysis without readmissions (Table S6). However, the day surgery pathway was still cost-saving (p<0.001) even when readmissions are factored in.

| Table S6: Generalized linear model results for bed-day costs approach with and without readmissions. | | | | |
| --- | --- | --- | --- | --- |
| Variable | **Cost ratio* without readmissions** | **P-value** | **Cost ratio**  **with readmissions** | **P-value** |
| Clinical pathway |  |  |  |  |
| Comparator group | Reference |  | Reference |  |
| Intervention Group | 0.37  (0.32-0.42) | <0.001 | 0.38  (0.33-0.46) | <0.001 |
|  |  |  |  |  |
| Sex |  |  |  |  |
| Female | Reference |  | Reference |  |
| Male | 0.79  (0.70-0.88) | <0.001 | 0.79  (0.70-0.88) | <0.001 |
|  |  |  |  |  |
| Age (years) | 1.01  (1.01-1.02) | <0.001 | 1.02  (1.01-1.02) | <0.001 |
| *Ratios are obtained by taking the exponent of the coefficient outputs of the regression model and can be interpreted as percentage changes. | | | | |

When the new coefficients (with readmissions) were used to calculate cost savings, the national savings decreased by £121,200 and the NOC savings decreased by £24,391 (Table S7).

| Table S7: Estimated cost savings per year using bed-day costs approach with and without readmissions. | | | |
| --- | --- | --- | --- |
|  | **Inpatient pathway** | **Day surgery pathway** | **Difference (net savings)** |
| *Bed-day costs approach without readmissions:* | | | |
| Mean bed-day cost per patient* | £1,432 | £527 | -£905 |
| No. of UKRs performed nationally** | 6060 | 6060 | - |
| Total national cost | £8,677,920 | £3,193,620 | **£5,484,300** |
| No. of UKRs performed at NOC*** | 434 | 434 | - |
| Total NOC cost | £621,488 | £228,718 | **£408,481** |
|  |  |  |  |
| *Bed-day costs approach with readmissions:* | | | |
| Mean bed-day cost per patient* | £1,431 | £546 | -£885 |
| No. of UKRs performed nationally** | 6060 | 6060 | - |
| Total national cost | £8,671,860 | £3,308,760 | **£5,363,100** |
| No. of UKRs performed at NOC*** | 434 | 434 | - |
| Total NOC cost | £621,054 | £236,964 | **£384,090** |
|  |  |  |  |
| *Estimate mean predicted by GLM model, controlling for age and gender.  **Number of UKRs performed in the United Kingdom (including England, Wales and Northern Ireland) in 2019 from the National Joint Registry.  **Actual number of UKRs performed in 2019 at the Nuffield Orthopaedic Centre. | | | |

Overall, the findings of the sensitivity analysis suggest that there are potential cost savings to be made from the day surgery pathway even when readmissions are considered, and complications and comorbidities are adjusted for.

**GBP to USD conversion:** 1 Great British Pound (£) = 1.25 US Dollar ($) (as of April 2024).

**Regression diagnostics**

In terms of model fit, Quantile-Quantile plots were used to conduct residual analysis of both OLS models and GLM models. For OLS models, residuals are expected to follow a normal distribution, which is shown on the Q-Q plot as the alignment of points on the diagonal line. For the GLM models, deviance residuals were used. Under the correct specification of the distribution function of the outcome variable (i.e. Poisson distribution for LOS and inverse Gaussian distribution for total costs), the points on the Q-Q plot also align on the diagonal line. As shown by the Figure S6, the GLM models had a better alignment of residuals than the OLS models, suggesting that the GLM models were generally a better fit for the data in this analysis. (Figure S3).

*Figure S3: Regression diagnostics: Ordinary Least Squares (OLS) regression vs Generalized Linear Model (GLM) regression.*


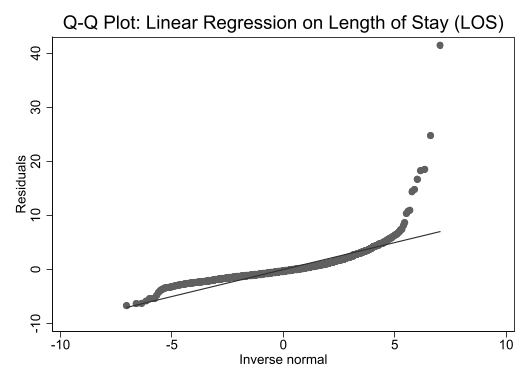

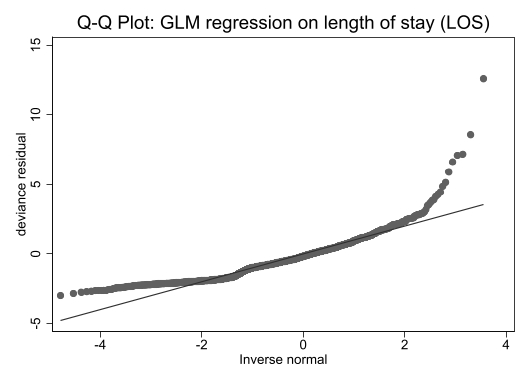


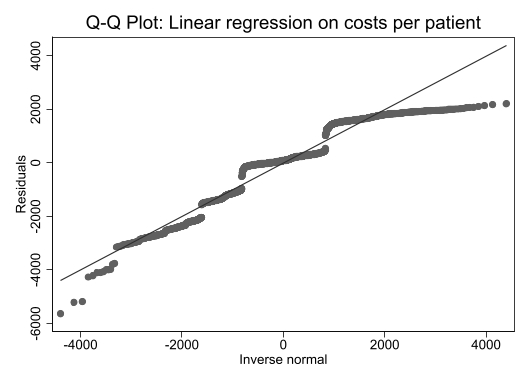

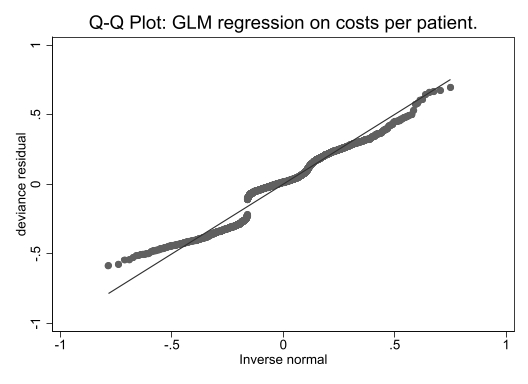


**REFERENCES**

[1] Jenkins C, Jackson W, Bottomley N, Price A, Murray D, Barker K. Introduction of an innovative day surgery pathway for unicompartmental knee replacement: no need for early knee flexion. Physiotherapy 2019;105(1):46-52. <https://doi.org/10.1016/j.physio.2018.11.305>.

[2] Jenkins C, Barker KL, Reilly KA, Pandit H, Dodd CAF, Murray DW. Physiotherapy management of minimally invasive Oxford medial compartment knee arthroplasty: an observational study of 100 patients following an accelerated treatment protocol. Physiotherapy 2006;92(4):214-8. <https://doi.org/10.1016/j.physio.2006.05.007>.

[3] 2021/22 National Cost Collection data; Available from: <https://www.england.nhs.uk/costing-in-the-nhs/national-cost-collection/>. [Accessed 21 June 2023].

[4] HRG4+ 2021/22 National Costs Grouper; Available from: <https://digital.nhs.uk/services/national-casemix-office/downloads-groupers-and-tools/hrg4-2021-22-national-costs-grouper>. [Accessed 13 July 2023].

[5] NHS Payment System 2023; Available from: <https://www.england.nhs.uk/pay-syst/national-tariff/>. [Accessed 4 August 2023].

[6] 2017/18 National Cost Collection Data; Available from: <https://webarchive.nationalarchives.gov.uk/ukgwa/20200501111106/https://improvement.nhs.uk/resources/reference-costs/>. [Accessed 30 July 2023].

[7] Jones KC. (2023) Unit Costs of Health and Social Care 2022 Manual; Available from: <https://kar.kent.ac.uk/100519/>.

[8] Murray DW, MacLennan GS, Breeman S, Dakin HA, Johnston L, Campbell MK, et al. A randomised controlled trial of the clinical effectiveness and cost-effectiveness of different knee prostheses: the Knee Arthroplasty Trial (KAT). Health Technol Assess 2014;18(19):1-235, vii-viii. <https://doi.org/10.3310/hta18190>.

[9] Belotti F, Deb P, Manning WG, Norton EC. Twopm: Two-Part Models. The Stata Journal 2015;15(1):3-20. <https://doi.org/10.1177/1536867x1501500102>.

[10] Glick HA, Doshi JA, Sonnad SS, Polsky D. Economic Evaluation in Clinical Trials*.* Oxford University Press; 2014.

[11] Hur ES, Serino J, Bohl DD, Della Valle CJ, Gerlinger TL. Fewer Adverse Events Following Outpatient Compared with Inpatient Unicompartmental Knee Arthroplasty. J Bone Joint Surg Am 2021;103(22):2096-104. <https://doi.org/10.2106/jbjs.20.02157>.

[12] Basques BA, Tetreault MW, Della Valle CJ. Same-Day Discharge Compared with Inpatient Hospitalization Following Hip and Knee Arthroplasty. J Bone Joint Surg Am 2017;99(23):1969-77. <https://doi.org/10.2106/jbjs.16.00739>.

[13] Bovonratwet P, Ondeck NT, Tyagi V, Nelson SJ, Rubin LE, Grauer JN. Outpatient and Inpatient Unicompartmental Knee Arthroplasty Procedures Have Similar Short-Term Complication Profiles. J Arthroplasty 2017;32(10):2935-40. <https://doi.org/10.1016/j.arth.2017.05.018>.
